# High-Dose Aspirin Reverses Tartrazine-Induced Cell Growth Dysregulation Independent of p53 Signaling and Antioxidant Mechanisms in Rat Brain

**DOI:** 10.1155/2019/9096404

**Published:** 2019-03-26

**Authors:** Nouf Alsalman, Abdulaziz Aljafari, Tanveer A. Wani, Seema Zargar

**Affiliations:** ^1^Department of Biochemistry, College of Science, King Saud University, Riyadh, Saudi Arabia; ^2^Department of Pharmaceutical Chemistry, College of Pharmacy, King Saud University, P.O. Box 2457, Riyadh 11451, Saudi Arabia

## Abstract

Tartrazine, an azo dye used in food, cosmetics, and pharmaceuticals with the effects on cell cycle, is not well understood. Therefore, we investigated the toxicity of tartrazine in rat brain with high-dose aspirin. Male Wistar rats (n = 24) were divided into (C) control, (T) tartrazine (700 mg/kg body weight [BW] at weeks 1 and 2), (A) aspirin (150 mg/kg [BW] at weeks 1, 2, and 3), and (TA) aspirin + tartrazine (150 mg/kg [BW] aspirin at weeks 1, 2, and 3 and 700 mg/kg [BW] tartrazine at weeks 1 and 2) groups. The expression of p53, B cell lymphoma-2 extra-large (Bcl-xL), cyclin-dependent kinase 2 (CDK2), p27, and Ki67 was evaluated by quantitative reverse-transcription PCR. A histopathological analysis of brain tissue and oxidative stress level was assessed based on reduced glutathione (GSH), ascorbic acid (AA), and malondialdehyde levels. We found that Bcl-xL, Ki67, CDK2, and p27 were upregulated and p53 was downregulated in the tartrazine-treated group as compared to the control group. Aspirin administration reversed these changes except P53 expression. Tartrazine had no effect on lipid peroxidation but altered AA and GSH levels with no reversal by aspirin treatment. Histopathological analysis revealed that aspirin prevented tartrazine-induced damage including increased perivascular space and hemorrhage. These results indicate that aspirin protects the brain from tartrazine-induced toxicity independent of p53 signaling and antioxidant mechanisms.

## 1. Introduction

Tartrazine (E 102, FD&C Yellow no. 5) is an artificially synthesized azo dye derived from coal tar; it is an orange-to-lemon-yellow-colored powder that is used in food, cosmetics, and pharmaceuticals. In some countries, tartrazine is used illegally as an alternative to saffron [1]. The recommended acceptable daily intake (ADI) of tartrazine is 7.5 mg/kg body weight (BW)/day [2]. Long-term intake or ingestion of tartrazine above the ADI can have adverse effects such as accelerating oxidative stress through the generation of reactive oxygen species (ROS) [1, 3]. Azo compounds have an azo (N=N) functional group with conjugated aromatic rings. The mutagenic, carcinogenic, and toxic effects of these compounds result from direct action or from the reductive biotransformation of the azo bond [4]. Orally ingested azo dye is mainly metabolized by azoreductase of intestinal microflora to aromatic amine, which is in turn oxidized to N-hydroxy derivatives by P450 enzymes [4]. Tartrazine reduction yields sulfanilic acid and aminopyrazolone, which can induce ROS generation and cause tissue damage [5]. Tartrazine is a neurotoxin that can cause a reduction in the volume of the medial prefrontal cortex, loss of neurons and glial cells, and a decrease in dendrite length [6]. Oral administration of tartrazine to male rat pups (500 mg/kg BW) altered the expression of antioxidant and oxidative stress markers and reduced neurotransmitters levels while increasing necrosis and apoptosis in the brain [1]. However, to date, there are no reports on the molecular basis of tartrazine toxicity [7].

Aspirin is a nonsteroidal anti-inflammatory drug that exerts its effects primarily by inhibiting cyclooxygenase enzymes [8]. Besides its analgesic, antipyretic, antiplatelet, and anti-inflammatory properties, aspirin is thought to be an antitumorigenic and neuroprotective agent [9]. Aspirin reduced neuroinflammation and oxidative stress in a rat model of neurocognitive disorder [8] and improved learning, memory, social behavior, and noncognitive behavior in mice; as such, it is recommended as a multitarget drug for Alzheimer's disease [10]. The antitumor activities of aspirin involve inhibition of cell proliferation by inducing cell cycle arrest through downregulation of cyclins and cyclin-dependent kinases (CDKs) and upregulation of CDK inhibitors [11]. Furthermore, high-dose aspirin was shown to prevent decreases in p53 expression in cancer and noncancer cell lines [12]. Other activities of aspirin include increased acetylation and activation of p53, leading to upregulation of the target genes p21 and B cell lymphoma (Bcl)-2-associated X protein, which causes cell cycle arrest and apoptosis, respectively [13]. Aspirin has also been reported to enhance apoptotic cell death by modulating the expression and activities of caspases and some Bcl-2 family members [14].

Based on the above observations, the present study evaluated the toxicity of tartrazine in rat brain and investigated whether high-dose aspirin can protect against neuronal damage caused by this compound.

## 2. Materials and Methods

### 2.1. Animals

Young male Wistar albino rats weighing 80–120 g (n = 24) were obtained from the Animal House Facility of King Saud University, Riyadh, Saudi Arabia. The study was approved by the animal ethics committee of King Saud University (approval no. KSU-SE-17-12). Rats were housed at 23°C–25°C and 55%–60% ambient humidity on a 12:12-hour light/dark cycle and were fed a normal diet with fresh drinking water daily.

### 2.2. Experimental Design

Tartrazine, aspirin, and phosphate-buffered saline (PBS) were purchased from Sigma-Aldrich Chemie GmbH (Darmstadt, Germany). Rats were randomly divided into the following four groups (n = 6 each): control (C); tartrazine (T; 700 mg/kg BW tartrazine for 2 weeks); aspirin (A; 150 mg/kg BW aspirin for 3 weeks); and aspirin + tartrazine (TA; 150 mg/kg BW aspirin 1 week before treatment with 700 mg/kg BW tartrazine and 150 mg/kg BW aspirin and 700 mg/kg BW of tartrazine for 2 weeks). Tartrazine was orally administered, whereas aspirin was intraperitoneally injected. Rats were sacrificed after treatments, and the brain was extracted and stored in 10% PBS at −80°C for assessment of oxidative stress or in RNAlater (Sigma-Aldrich Chemie GmbH) at −80°C for RNA extraction.

### 2.3. RNA Extraction and cDNA Preparation

Total RNA was extracted from brain tissue using trizol reagent (Sigma-Aldrich Chemie GmbH) according to manufacturer's instructions. cDNA was prepared using the Superscript III first strand synthesis system (Invitrogen, Carlsbad, CA, USA) according to the manufacturer's instructions.

### 2.4. Quantitative Polymerase Chain Reaction (qPCR) Analysis

Ki67, CDK2, Bcl-2 extra-large (Bcl-xL), p53, and p27 transcripts were detected by qPCR on a ViiA7 High Productivity Real-Time PCR System (Thermo Fisher Scientific, Waltham, MA, USA). Primers were designed from exon to exon junction using Primer 3 software (https://primer3plus.com/cgi-bin/dev/primer3plus.cgi) and were synthesized by Metabion International AG (Planegg, Germany); the sequences are shown in Table 1. PCR was performed using SYBR Green ROX qPCR Master Mix (Qiagen, Hilden, Germany), with glyceraldehyde 3-phosphate dehydrogenase (GAPDH) used as the reference gene. Each 20 *μ*l reaction mixture contained equal quantities of cDNA, 5 *μ*M each primer, and 10 *μ*l Master Mix. Cycling conditions were similar to those reported in our previous study [15]. mRNA levels were calculated and are presented as fold change relative to GAPDH.

### 2.5. Measurement of Oxidative Stress

Brains were weighed using an analytic balance (Mettler-Toledo, Columbus, OH, USA) and homogenized in 10% w/v PBS using an electric homogenizer (PRO Scientific, Oxford, CT, USA); the debris was removed by centrifugation (Thermo Fisher Scientific) at 2000 ×* g* for 10 min at 4°C. The homogenate was used to assess malondialdehyde (MDA), reduced glutathione (GSH), and ascorbic acid (AA) levels. Lipid peroxidation level was determined by measuring the concentration of MDA with the thiobarbituric acid (British Drug Houses, London, UK) reaction [16]. The level of GSH was estimated [17] by reacting with 5,5′-dithio-bis-[2-nitrobenzoic acid (Sigma-Aldrich Chemie GmbH), which yields a yellow-colored product that absorbs light at 412 nm. AA concentration was determined by a previously described method [18] using Folin reagent (Sigma-Aldrich Chemie GmbH), which produces a blue-colored product that absorbs light at 760 nm. AA and GSH concentrations were extrapolated from standard curves. All values are expressed per gram of tissue.

### 2.6. Statistical Analysis

Data are expressed as mean ± standard error and were analyzed using Prism v.7.3 (GraphPad Inc., La Jolla, CA, USA) and Excel (Microsoft, Redmond, WA, USA) software. Fold changes in gene expression were calculated with the 2^−ΔΔCT^ method in Excel. Means were compared by one-way analysis of variance followed by Tukey's multiple comparison test with a 95% confidence interval. A P value ≤ 0.05 was considered significant.

## 3. Results

### 3.1. Expression of Genes Associated with Cell Proliferation and Cell Cycle Regulation

**Figure 1:**
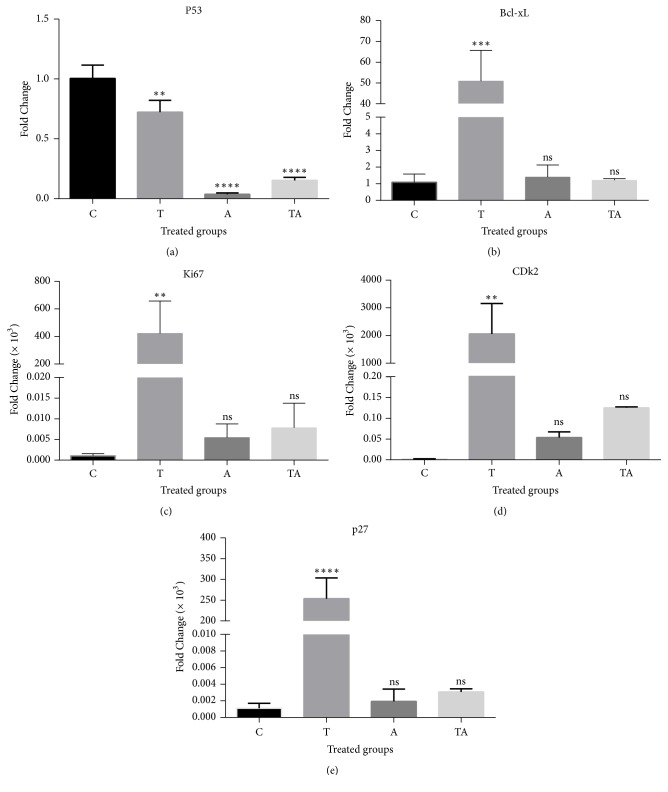
Relative changes in gene expression in the brain of rats in the treatment groups relative to the control group. (a–e) Fold changes in p53 (a), Bcl-xL (b), Ki67 (c), CDK2 (d), and p27 (e) are shown. Data are expressed as mean ± SE. ^*∗*^P < 0.05, ^*∗∗*^P < 0.01, and ^*∗∗∗*^P < 0.001 versus control; ns, nonsignificant. A, 150 mg/kg aspirin; C, control; T, 700 mg/kg tartrazine; TA, tartrazine + aspirin.

The mRNA level of p53 was decreased in all treatment groups relative to the control. The fact that the levels in the A and TA groups were similar indicated that the effects of tartrazine are independent of p53 (Figure 1). Bcl-xL expression was increased significantly in the T group, whereas the increase in the A and TA groups was insignificant compared to the control. Ki67, CDK2, and p27 expressions were significantly upregulated in the T group, whereas in the A and TA groups the upregulation was insignificant relative to the control.

### 3.2. Histopathological Analysis of Brain Tissue

**Figure 2:**
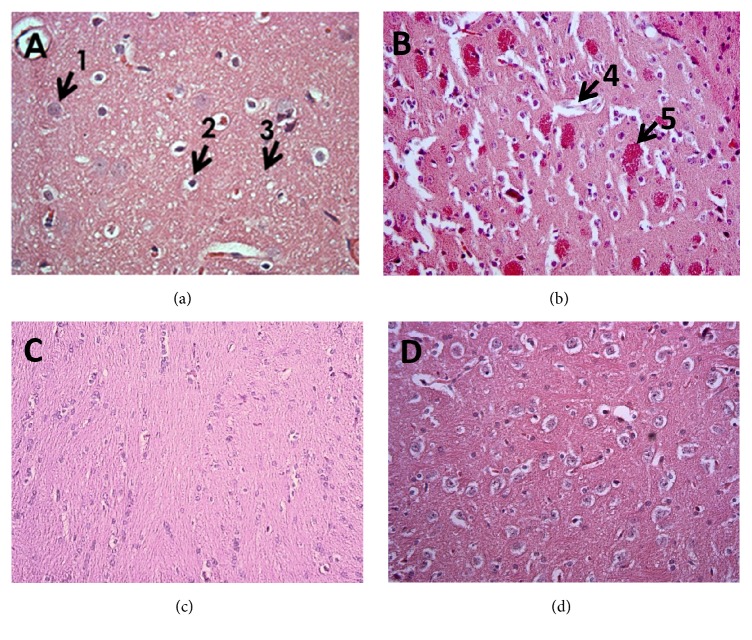
Histopathological analysis of brains of rats in treatment and control groups. (a–d) Representative brain sections from control rats with normal intact tissue (a) and rats treated with 700 mg/kg body weight tartrazine (b), 150 mg/kg body weight aspirin (c), and tartrazine + aspirin (d) are shown. 1, neuron; 2, glial cell; 3, neuropil; 4, perivascular space; 5, hemorrhage.

An examination of hematoxylin/eosin-stained brain tissue sections by microscopy revealed an increase in the perivascular space with hemorrhage in rats treated with tartrazine. In contrast, rats in the A and TA groups showed no obvious changes in the brain relative to control animals, with intact tissue and normal neurons and glial cells (Figure 2).

### 3.3. Oxidative Stress in the Brain

**Figure 3:**
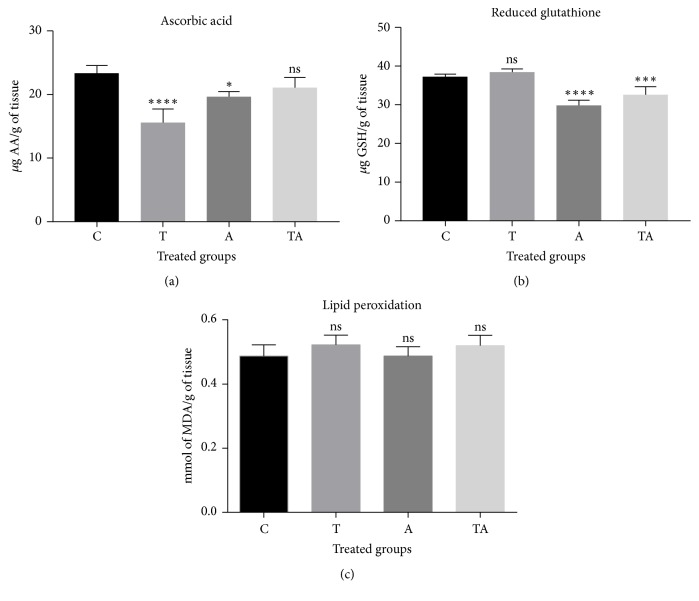
Oxidative stress in brains of rats in the treatment and control groups. (a–c) Changes in AA (a) and GSH (b) concentrations and lipid peroxidation level (c) were evaluated at the end of the experimental period. Data are expressed as mean ± SE. ^*∗*^P < 0.05 versus control; ns, nonsignificant. A, 150 mg/kg aspirin; C, control; T, 700 mg/kg tartrazine; TA, tartrazine + aspirin.

Tartrazine decreased AA level in the brain (15.673 *μ*g/g tissue) compared to the control group (23.29 *μ*g/g tissue) (Figure 3). The level was also decreased in the TA group, but the difference relative to the control was nonsignificant (21.124 *μ*g/g tissue). GSH concentration did not differ significantly between the T group (38.631 *μ*g/g of tissue) and control rats (37.45 *μ*g/g tissue) but was lower in the A and TA groups (29.05 and 32.9 *μ*g/g tissue, resp.). The degrees of lipid peroxidation—as measured by MDA level—in the brain were similar across groups (0.556, 0.494, 0.486, and 0.5 mmol/h g tissue for T, A, TA, and C groups, resp.).

## 4. Discussion

Artificial colors added to food can have harmful and toxic effects [1]. Tartrazine is reportedly toxic, whereas aspirin is thought to have prophylactic effects at low doses [8, 10, 19]. In this study, we investigated the effects of the color additive tartrazine on the expression of cell cycle regulatory genes, oxidative stress, and histopathological alterations in the brain and examined whether high-dose aspirin would protect against these effects.

All but one of the examined genes (p53) were dysregulated by tartrazine, although normal expression was restored by aspirin treatment. These results indicate that high-dose aspirin reverses the negative effects of tartrazine on cell growth via a mechanism that is independent of p53 signaling. p53 has dichotomous roles, with overexpression and deficiency both leading to disease [20]. A decrease in p53 transcript can result from feedback inhibition by p53 protein [21], which can accumulate due to proteasome inhibition in response to injury, with a consequent reduction in protein clearance [22, 23]. High doses of aspirin were shown to prevent the downregulation of p53 in cancer and noncancer cell lines and in mouse liver by preventing mouse double minute 2 homolog-mediated proteasomal degradation of p53 [12].

Bcl-xL mRNA expression was increased in the T group. Overexpression of Bcl-2 and other antiapoptotic proteins is common in many cancers [24]. Bcl-xL overexpression was reported in primary human breast carcinoma and some breast cancer cell lines [25]. Elevated expression of the antiapoptotic proteins Bcl-2 and Bcl-w in human malignant glioblastoma and decreased expression of proapoptotic bcl-2 family members inhibit the apoptosis of tumor cells [26]. Previous studies have reported conflicting findings with respect to Bcl-xL expression in the context of tartrazine genotoxicity/carcinogenicity [7, 8, 27–29]. The upregulation of Bcl-xL observed in this study may indicate impaired p53 signaling, leading to apoptosis resistance, enhanced cell proliferation, and escape from cell cycle arrest in the brain following tartrazine administration.

In the present study, the T group showed a marked increase in the mRNA levels of the proliferation marker Ki67, CDK2, and the cyclin-dependent kinase inhibitor p27. CDK2 is a serine/threonine protein kinase that regulates the G1/S transition, DNA synthesis, and exit from S phase. CDK2 is activated in the late G1 and S phases by forming a complex with cyclin E and cyclin A, respectively [30]. p21 and p27 can interact to block the kinase activities of these two complexes [30, 31], and p21 can arrest the cell cycle in G1 phase by inhibiting the cyclin E/CDK complex, thereby repressing the transcription of genes required for progression to S phase [32, 33]. In this study, p27 overexpression was accompanied by upregulation of Ki67, indicating a high level of proliferation in the T group. Overexpression of p27 in some breast carcinomas is correlated with lymph node metastasis [34] and tumor progression [35]; thus, the increased expression of Ki67 induced by tartrazine in this study may reflect the development of glioma. The level of p53 genes is being downregulated (as compared to control), while other genes are upregulated. This result is concomitant with previous studies that reported decreased expression of P53 on different types of stresses. p53 acts as a transcription factor that activates or represses many gene clusters [36]. p53 in many stresses induces its own inhibitor Mdm2, forming a negative autoregulatory loop in messenger RNA, which forces degradation of p53 mRNA, as Mdm2 activity increases [37].

Histopathological changes in the brain of tartrazine-treated rats included an enlarged perivascular space and hemorrhage. The former is associated with increased arterial pressure [38]; indeed, one of the harmful effects of tartrazine is the induction of hypertension. Our results are consistent with previous studies in which intravenous injection of tartrazine (0.1–2.0 mg/kg) increased mean arterial blood pressure [39, 40]. However, the brain tissue of rats in the A and TA groups was intact, providing additional evidence for the neuroprotective effects of aspirin [41].

In the present study, administration of tartrazine (700 mg/kg BW) for 2 weeks decreased the level of AA relative to control animals; this was reversed by pretreatment with aspirin, but rats treated with aspirin only also showed a significant difference in AA level compared to the control. Furthermore, tartrazine treatment did not alter brain GSH and MDA levels. Many previous studies have reported that tartrazine administration for 30 consecutive days increased ROS production, leading to oxidative damage [4, 5, 42–44]). The discrepancy between these earlier studies and ours may be due to a difference in duration of the experiment; it is possible that the antioxidant defense system of the brain was able to withstand tartrazine-induced oxidative stress during the 14-day treatment in this study. An alternative explanation is that lipid peroxidation only occurs in the late stages of oxidative stress [45] and that the markers we examined did not reflect the full effects of tartrazine.

In summary, the results of this study demonstrate that tartrazine is genotoxic and potentially tumorigenic in the brain. High doses of aspirin can serve as a prophylactic agent to mitigate these effects. Our findings provide novel insight into the mechanism of action of tartrazine and evidence that aspirin can prevent damage to the brain caused by this widely used synthetic dye.

## Figures and Tables

**Table 1:** Sequences of primers for qPCR analysis.

Transcript	Forward primer (5′→3′)	Reverse primer (5′→3′)
Ki67	AAGAAGAGCCCACAGCACAGAGAA	AAGAAGAGCCCACAGCACAGAGAA
CDK2	TTCTGCCATTCTCACCGTGTCCTT	TGCGATAACAAGCTCCGTCCATCT
Bcl-xL	AGAGAGGCAGGCGATGAGTTTGAA	TCCAACTTGCAATCCGACTCACCA
p53	AACAATGGCCCGAGTCTAATGGGA	ACAGATGTTGCCTGATGTCTGGGT
p27	ATATGGAAGAAGCGAGTCAGCGCA	ACGAACCTCTGGGAAATGGGTTCT
GAPDH	ACCACAGTCCATGCCATCAC	ACCACAGTCCATGCCATCAC

## Data Availability

The data can be reproduced on demand.

## Abbreviations

AA: Ascorbic acid
ADI: Acceptable daily intake
Bcl-xL: B cell lymphoma-2 extra-large
CDK2: Cyclin-dependent kinase 2
GAPDH: Glyceraldehyde 3-phosphate dehydrogenase
PBS: Phosphate-buffered saline
ROS: reactive oxygen species.

## Supplementary Materials

Supplementary Materials The supplementary file includes table containing C_t_ values of the target genes of all the groups: malondialdehyde, reduced glutathione, and ascorbic acid level in the rat brain of control and treated groups.
