# Supplementary material for: High-Dose Aspirin Reverses Tartrazine-Induced Cell Growth Dysregulation Independent of p53 Signaling and Antioxidant Mechanisms in Rat Brain
Source: Biomed Res Int. 2019 Mar 26;2019:9096404. doi: 10.1155/2019/9096404 (PMC6457281; doi:10.1155/2019/9096404)
Supplement: Supplementary Materials — The supplementary file includes table containing Ct values of the target genes of all the groups: malondialdehyde, reduced glutathione, and ascorbic acid level in the rat brain of control and treated groups. [file 9096404.f1.zip › 9096404.f1/9096404_SupplDesc.docx]

The supplementary file includes, Table containing Ct values of the target genes of all the groups; Melanoaldahyde, reduced glutathione, ascorbic acid level in the rat brain of control and treated groups.
