# Supplementary material for: High-Dose Aspirin Reverses Tartrazine-Induced Cell Growth Dysregulation Independent of p53 Signaling and Antioxidant Mechanisms in Rat Brain
Source: Biomed Res Int. 2019 Mar 26;2019:9096404. doi: 10.1155/2019/9096404 (PMC6457281; doi:10.1155/2019/9096404)
Supplement: Supplementary Materials — The supplementary file includes table containing Ct values of the target genes of all the groups: malondialdehyde, reduced glutathione, and ascorbic acid level in the rat brain of control and treated groups. [file 9096404.f1.zip › 9096404.f1/mat.9096404.v2.docx]

**Table 1:** C_t_ values of the target genes of all the groups:

| **Gene** | **Ct valus** | | | |
| --- | --- | --- | --- | --- |
|  | **Control group** | **Tartrazine group** | **Aspirin group** | **Aspirin with Tartrazine group** |
| ***p53*** | 19.17 | 36.639 | 26.891 | 24.091 |
|  | 18.85 | 36.836 | 26.961 | 23.637 |
|  | 19.005 | 37.034 | 26.166 | 23.703 |
| ***BCL-xl*** | 17.900 | 28.931 | 20.913 | 18.940 |
|  | 17.210 | 28.507 | 19.21 | 18.954 |
|  | 16.855 | 29.355 | 19.161 | 19.152 |
| ***CDK2*** | 37.345 | 36.335 | 33.172 | 31.274 |
|  | 34.942 | 33.581 | 32.942 | 31.253 |
|  | 36.143 | 31.936 | 33.693 | 31.229 |
| ***Ki67*** | 33.062 | 32.433 | 32.551 | 33.786 |
|  | 32.135 | 31.723 | 33.205 | 31.105 |
|  | 33.32 | 30.81 | 34.608 | 32.053 |
| ***p27*** | 33.679 | 33.679 | 35.473 | 35.148 |
|  | 34.413 | 34.413 | 37.819 | 34.796 |
|  | 35.31 | 35.31 | 36.646 | 34.859 |
| ***GAPDH*** | 11.71 | 27.558 | 13.631 | 13.372 |
|  | 11.189 | 29.970 | 15.589 | 13.574 |
|  | 11.385 | 28.779 | 13.573 | 13.572 |

**Table 2:** The Melanoaldahyde level in the rat brain of all the treated groups.

| **Group** | **Control** | **Tartrazine** | **Aspirin** | **Aspirin with Tartrazine** |
| --- | --- | --- | --- | --- |
| **Melanoaldahyde level (mmol/g tissue)** | 0.45 | 0.56 | 0.45 | 0.50 |
|  | 0.53 | 0.53 | 0.49 | 0.56 |
|  | 0.47 | 0.51 | 0.52 | 0.49 |
|  | 0.50 | 0.49 | 0.49 | 0.53 |

**Table 3:** The reduced glutathion level in the rat brain of all the treated groups.

| **Group** | **Control** | **Tartrazine** | **Aspirin** | **Aspirin with Tartrazine** |
| --- | --- | --- | --- | --- |
| **GSH concentration (µg/ g tissue)** | 37.450 | 38.850 | 25.200 | 32.900 |
|  | 37.800 | 37.800 | 31.500 | 33.600 |
|  | 36.750 | 39.375 | 29.050 | 30.450 |
|  | 37.800 | 38.500 | 30.450 | 34.650 |

**Table 4:** The ascorbic acid level in the rat brain of all the treated groups.

| **Group** | **Control** | **Tartrazine** | **Aspirin** | **Aspirin with Tartrazine** |
| --- | --- | --- | --- | --- |
| **Ascorbic acid concentration (µg/ g tissue)** | 22.230 | 12.791 | 19.186 | 21.124 |
|  | 25.000 | 16.860 | 19.186 | 20.349 |
|  | 23.256 | 15.600 | 20.349 | 19.767 |
|  | 22.674 | 17.442 | 20.349 | 23.256 |
